# Effect of empagliflozin on weight in patients with prediabetes and diabetes

**DOI:** 10.1038/s41598-024-83820-7

**Published:** 2025-01-02

**Authors:** Mojgan Sanjari, Mohammad Hadavizadeh, Narges Sadeghi, Ahmad Naghibzadeh-Tahami

**Affiliations:** 1 Endocrinology and Metabolism Research Center, Institute of Basic and Clinical Physiology Sciences, Kerman University of Medical Sciences, Kerman, Iran; 2 Physiology Research Center, Institute of Neuropharmacology, Kerman University of Medical Sciences, Kerman, Iran; 3 Health Services Management Research Center, Institute for Futures Studies in Health, Kerman University of Medical Sciences, Kerman, Iran

**Keywords:** Empagliflozin, Weight, Waist circumference, Body mass index, Diabetes, Prediabetes, Obesity, Diabetes, Diabetes complications, Type 2 diabetes

## Abstract

The impact of blood glucose-lowering medications on weight has always been a topic of interest in the treatment of diabetic patients. This study investigates the effect of empagliflozin on weight in patients with prediabetes and type 2 diabetes. This quasi-experimental study was performed on patients with prediabetes or type 2 diabetes with an HbA1c level up to 1% higher than the treatment target, and not using other blood glucose-lowering medications. Patients received 10 milligrams of Empagliflozin once daily for three months, and weight, BMI, waist circumference, and blood pressure were evaluated monthly. Forty-three patients (21 women and 22 men) enrolled. The average weight of patients decreased by 2.96 ± 1.96 kg (3.8%) (*P* < 0.001). BMI decreased by -1.10 ± 0.71 Kg/m^2^ (3.72%) (*P* < 0.001). The waist circumference of patients decreased by -3.23 ± 3.69 centimeters (*P* < 0.001). FPG decreased from 114.86 to 109.48 mg/dL (*P* < 0.001), and HbA1c decreased from 6.52% to 6.38% (*P* < 0.001). The weight change was more significant in men than women (-3.59 ± 1.74 vs. -2.30. ± 1.99), (*P* = 0.029). Weight reduction was greater in patients with GFR higher than 90 compared to GFR lower than 90 (-3.34. ± 2.00 vs. -2.16 ± 1.67) (*P* = 0.063). Also, no significant difference was observed in the weight, BMI, and waist circumference changes between different BMI groups (less than 25, 25 to 30, 30 to 35, and higher than 35). The trend of weight and BMI changes during the three-month empagliflozin treatment period was significant (*P* < 0.001) and didn’t reach a plateau level after three months. The change in waist circumference was also significant, reaching a plateau level after one month (*P* < 0.001). There was no significant change in blood pressure. In conclusion the weight, BMI, and waist circumference of patients decreased following the administration of empagliflozin. Weight reduction was more pronounced in males than females and in patients with a GFR above 90 than those with a GFR below 90. *Trial registration*: This study was approved by the Research Deputy of Kerman University of Medical Sciences with tracking number 401,000,516, IRCT code: IRCT20090317001774N10, and ethical code: IR.KMU.AH.REC.1402.065 was approved by Research Ethics Committee of Afzalipour Hospital- Kerman University of Medical Sciences.

## Background

“Diabetes mellitus (DM) refers to a group of common metabolic disorders that share the phenotype of hyperglycemia.”^1^ Chronic elevation of blood glucose levels can lead to severe damage to the heart, blood vessels, kidneys, eyes, and nerves. According to the World Health Organization (WHO), approximately 422 million people worldwide are affected by diabetes, and 1.5 million people die directly due to diabetes each year^2^.

The prevalence of Diabetes mellitus has significantly increased in recent years, with the global prevalence of diabetes rising from 108 million in 1980 to 422 million in 2014^3^. According to the WHO’s report, the prevalence of diabetes in Iran was 10.3% in 2016, with 11.1% of women and 9.6% of men affected. Obesity and overweight are risk factors for diabetes^4^.

A study conducted in 2021 by Najafi pour et al. in Kerman, a province in Iran, reported the prevalence of individuals with prediabetes, diagnosed diabetes, and undiagnosed diabetes as 12% (13.2% in men and 11.1% in women), 10.3% (8% in men and 11.8% in women), and 1.9%, respectively^5^.

Overweight, obesity, and a lack of physical activity are preventable risk factors for diabetes^6^. In 2016, 60.5% of the Iranian population was overweight (63.1% of women and 58.0% of men), and 24.9% had obesity (30.6% of women and 19.3% of men)^4^. Obesity is a significant public health concern of the modern era and is independently associated with cardiovascular events and stroke^7^. Additionally, obesity is recognized as one of the complications of type 2 diabetes. Even modest weight loss can significantly improve glucose homeostasis and reduce metabolic cardiovascular risk factors in type 2 diabetes patients. However, long-term lifestyle-based weight reduction strategies are not very effective. There is a growing need to consider pharmacological approaches to aid in weight reduction in diabetic patients^8^.

Various medications are used in the treatment of diabetes. One important class of diabetes drugs is Sodium-glucose Cotransporter-2 (SGLT2) Inhibitors, which inhibit the simultaneous reabsorption of glucose and sodium in the proximal tubules of the kidneys, leading to urinary glucose excretion. Some drugs in this group include Canagliflozin, Dapagliflozin, and Empagliflozin. These drugs reduce weight and blood pressure, lower the risk of cardiovascular events, have a renal protective effect and do not cause hypoglycemia. However, they have disadvantages, such as an increased risk of urinary and genital tract infections, dehydration, polyuria, and a heightened tendency for hyperkalaemia and diabetic ketoacidosis^1^.

As mentioned, diabetes and obesity are two major health problems. Furthermore, weight reduction can enhance the effectiveness of insulin. Therefore, assessing the impact of effective drugs on weight in the diabetic population in Kerman is essential due to the varying effects of drugs on weight in different populations and the limited studies conducted within Iran.

Although the benefits of Empagliflozin, such as weight reduction, are well recognized, most studies have primarily assessed its effectiveness as a second-line treatment in combination with Metformin. Moreover, most studies have been conducted on patients with uncontrolled diabetes or heart failure. In contrast, our study investigates the effects of Empagliflozin as a first-line therapy in patients with prediabetes or diabetes with HbA1c levels up to 1% above the diagnostic threshold. This semi-experimental study over 12 weeks also examines the trends in weight, waist circumference, and body mass index (BMI) changes every month. We compare the changes in weight, waist circumference, and BMI among genders, glomerular filtration rate (GFR), and BMI groups.

## Methods

This study is a semi-experimental clinical trial with a before-and-after design conducted over 12 weeks on 43 participants (22 males and 21 females).

The study population consisted of individuals aged 18 to 65 who were diagnosed with prediabetes or confirmed diabetes mellitus and had either visited one of the clinics affiliated with the Kerman University of Medical Sciences in 2022 or 2023 or were referred from the Kerman University of Medical Sciences Cohort Study (KERCADR). This study commenced on September 22, 2022, and concluded on April 21, 2023.

The non-probability convenience sampling method was used to reach the desired sample size. After obtaining the necessary approvals from the regulatory authorities of Kerman University of Medical Sciences, eligible individuals were selected based on entry and exit criteria, provided with an explanation of the study procedures, and obtained written informed consent (using an informed consent form) for participation.

The inclusion criteria for participants in the study were having prediabetes or diabetes with an HbA1c level of no more than 1% above the target for diabetes management and not receiving blood sugar-controlling medications.

Prediabetes and diabetes were defined based on criteria provided by the American Diabetes Association, where one of the following conditions was equivalent to prediabetes or diabetes:

- Fasting blood glucose levels between 100 and 125 mg/dL is equivalent to prediabetes, or greater than 125 mg/dL is equal to diabetes.
- 2-hour laboratory blood glucose level (2 h after consuming 75 g of oral glucose) between 140 and 199 mg/dL is equivalent to prediabetes or equal to or higher than 200 mg/dL is equivalent to diabetes.
- Random laboratory blood glucose levels equal to or above 200 mg/dL are equivalent to diabetes.
- HbA1c level between 5.7% and 6.4% is equivalent to prediabetes, or equal to or higher than 6.5% is equivalent to diabetes.

The exclusion criteria for participants from the study included:

- Diagnosis of type 1 diabetes mellitus.
- Use of injectable or oral blood sugar-controlling medications (insulin, biguanides, sulfonylureas, thiazolidinediones, sodium-glucose cotransporter 2 (SGLT2) inhibitors, GLP-1 receptor agonists, bromocriptine, or cholestyramine).
- History of diabetic ketoacidosis.
- Glomerular filtration rate (GFR) less than 30.
- Underlying conditions predisposing to acidosis.
- Liver failure.

After participants’ enrolment, demographic and personal information, including weight (measured using a calibrated scale accurate to 0.1 kg from the brand Seca), height, waist circumference, systolic and diastolic blood pressure (measured after 15 min of sitting in the clinic), were recorded according to a data extraction form. Subsequently, all participants received a daily dose of 10 mg of empagliflozin (manufactured by Dr. Abidi, Iran, under the trade name Gloripa) for three months, and their weight, waist circumference, systolic and diastolic blood pressure, and potential side effects were monitored monthly. Finally, changes in these parameters during these three months as a result of drug consumption were compared and analysed.

Statistical analysis was performed using IBM SPSS Statistics 27 software. During the data preparation process, one participant was excluded from the study due to intentional weight loss. Patients’ characteristics were calculated using means. The initial and final study data were compared using the Paired-Samples T-test, and the magnitude of changes throughout the study was calculated. Changes based on gender and GFR were assessed using the Independent Sample T-test, and the size of modifications based on BMI was calculated using the ANOVA One-Way statistical test. Out of the 43 patients, 36 attended all four scheduled visits, and data concerning these participants were utilized to analyse monthly trends in weight, body mass index, and waist circumference. The Repeated Measures Test was employed to explore the monthly patterns of change. Finally, Generalized Estimating Equations were utilized to predict the relationship between the mean weight and other variables.

This study was approved by the Research Deputy of Kerman University of Medical Sciences with tracking number 401,000,516, IRCT code: IRCT20090317001774N10, Registration date: 23/02/2023, and ethical code: IR.KMU.AH.REC.1402.065 and was approved by the Research Ethics Committee of Afzalipour Hospital- Kerman University of Medical Sciences and all methods were performed in accordance with the relevant guidelines and regulations.

## Results

**Fig. 1:**
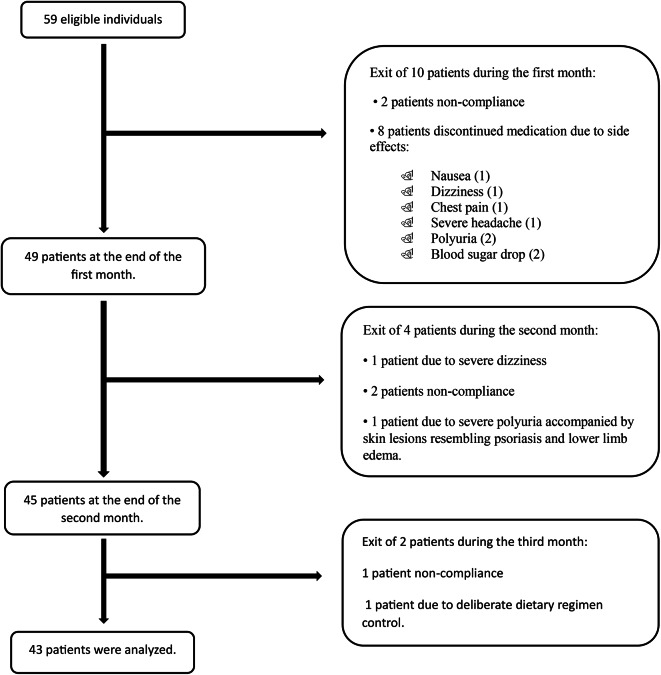
Study design flowchart.

Forty-three participants (21 females and 22 males) completed the study. (Refer to Fig. 1 for the Study Design Flowchart) The average age of the participants was 54.84 ± 7.98 years.

**Table 1:** Changes in patients’ characteristics from baseline.

variables	0 weeks	12 weeks	*P* value
Body weight (kg)	77.3(16.0)	74.3(15.1)	0.001
Waist circumference (cm)	101.5(9.3)	98.3(9.4)	0.001
BMI (kg/m^2^)	29.5(4.2)	28.4(4.0)	0.001
SBP (mmHg)	136.4(15.5)	132.4(18.4)	0.069
DBP (mmHg)	82.5(10.3)	83.7(9.7)	0.170
eGFR (mg/ml/1.73m^2^)	107.6(31.1)	94.3(25.9)	0.001
FPG (mg/dL)	114.86(28.9)	109.48(21.7)	0.001
HbA1c (%)	6.52(0.68)	6.38(0.76)	0.001

According to Table 1, after 12 weeks of empagliflozin treatment, there was a significant reduction in body weight, BMI, and waist circumference. Weight changes ranged from + 1.8 to -6.3 kg, and BMI changes ranged from + 0.83 to -2.23 kg/m². No significant differences were observed in blood pressure measurements. Additionally, there were significant reductions in HbA1c and Fasting plasma glucose (FPG).

**Table 2:** Change from baseline.

variables	Male	Female	*P* value	GFR > 90	GFR < 90	*P* value	BMI < 25	25 < BMI < 30	30 < BMI < 35	35 < BMI
Body Weight(kg)	-3.59 ± 1.74	-2.30 ± 1.99	0.029	-3.34 ± 2.00	-2.16 ± 1.67	0.063	-2.01 ± 2.35	-2.92 ±1.70	-2.92 ±2.04	-4.34 ± 1.89
Waist circumference(cm)	-4.20 ± 3.40	-2.21 ± 3.80	0.077	-3.63 ± 4.03	-2.39 ± 2.84	0.307	-3.25 ±5.52	-3.79 ±2.03	-2.90 ±4.90	-2.30 ± 1.30
BMI(kg/m^2^)	-1.23 ± 0.59	-0.91 ± 0.81	0.231	-1.22 ± 0.72	-0.87 ± 0.66	0.131	-0.70 ± 0.80	-1.15 ±0.64	-1.05 ±0.74	-1.59 ± 0.62

According to Table 2, The weight loss was greater in men than women, but no significant differences were observed in the BMI or waist circumference changes between the two groups.

To investigate the mean changes in demographic variables based on the glomerular filtration rate (GFR) level of the patients, we divided them into two groups: those with GFR less than 90 and those with GFR greater than 90. The weight reduction was more pronounced in individuals with a GFR above 90 compared to those with a GFR below 90. This difference was relatively statistically significant (*P* = 0.063). However, the two groups found no significant differences in the BMI or waist circumference changes.

To investigate the average changes in demographic variables based on the patients’ body mass index (BMI), they were divided into four groups according to BMI at the beginning of the study (less than 25, between 25 and 30, between 30 and 35, and above 35). there were no significant differences in weight, BMI, or waist circumference changes among the different BMI groups.

Among the initial 43 patients, 36 patients completed all four scheduled visits. Changes in demographic variables were analyzed monthly using the Repeated Measures Test for these 36 patients across four visits: the baseline visit, the second visit (one month after initiating Empagliflozin treatment), the third visit (two months after initiation), and the fourth visit (three months after initiation).

**Fig. 2:**
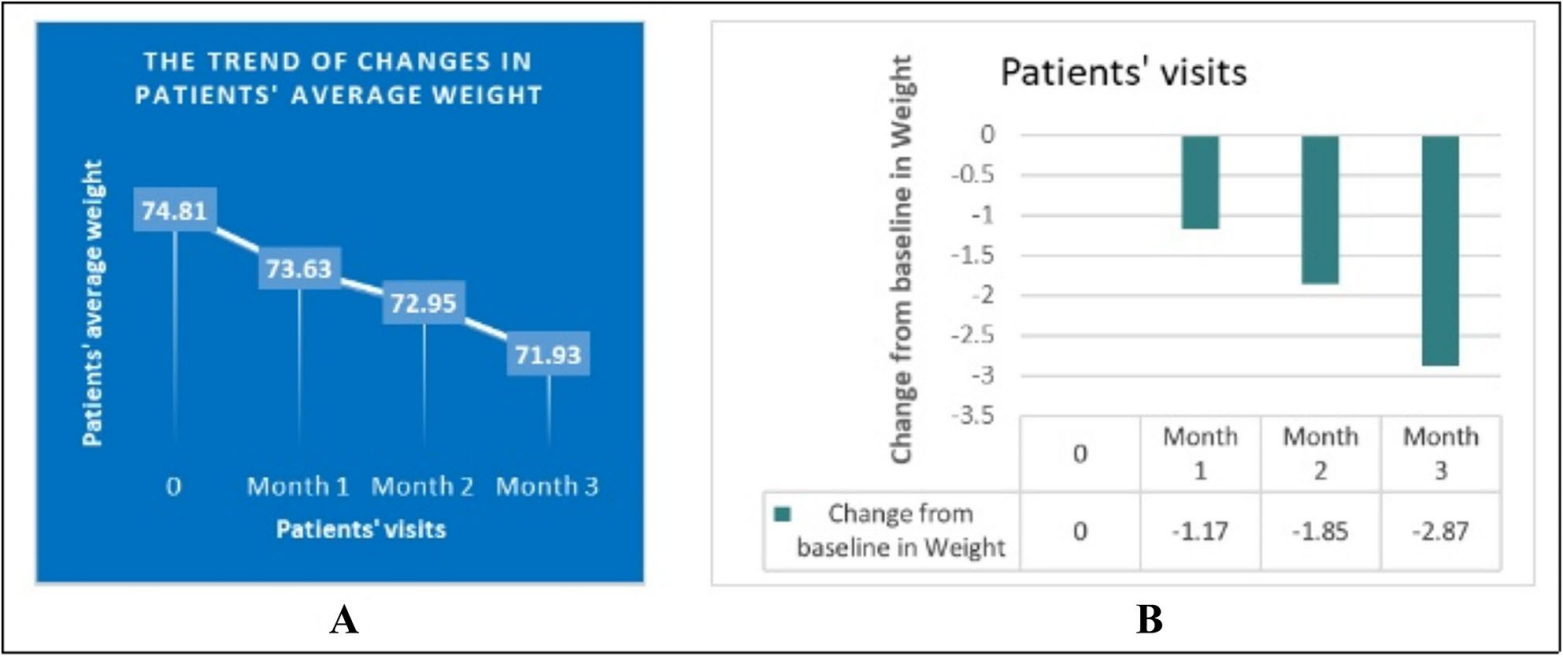
(**A**) The trend of changes in patients’ average weight. (**B**) Change from baseline in weight. Difference between baseline and first month =-1.17 Kg (*P* value < 0.001). Difference between first and second month =-0.67 Kg (*P* value < 0.001). Difference between second and third month =-1.022 Kg (*P* value < 0.001).

According to Fig. 2, the average weight of patients at the baseline, first, second, and third month was 74.80 ± 2.11, 73.63 ± 2.05, 72.95 ± 2.00, and 71.93 ± 1.94, respectively, and the difference in average weight between patient visits was statistically significant (*P* < 0.001).

**Fig. 3:**
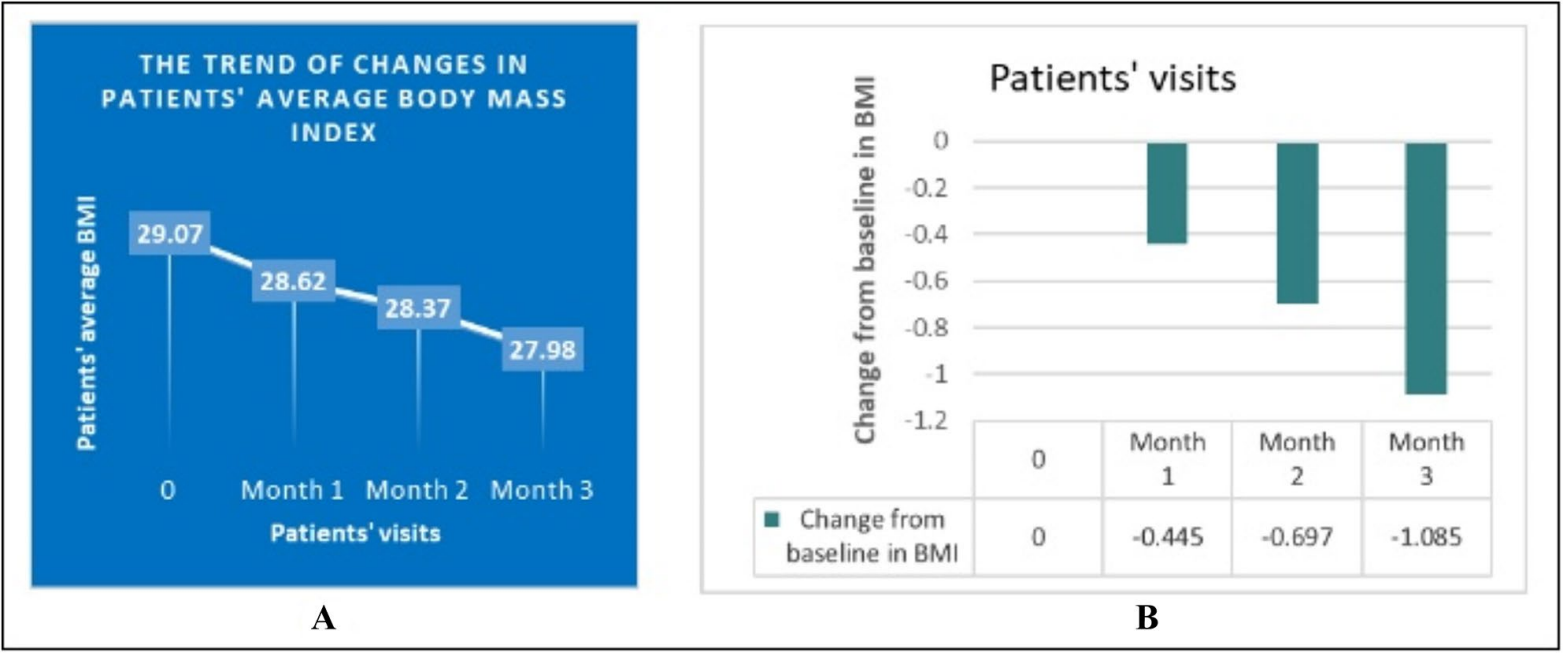
(**A**). The trend of changes in patients’ average body mass index. (**B**) Change from baseline in body mass index.. Difference between baseline and first month =-0.445 Kg/m^2^ (*P* value < 0.001). Difference between first and second month =-0.252 Kg/m^2^ (*P* value = 0.002). Difference between second and third month =-0.388 Kg/m^2^ (*P* value < 0.001).

According to Fig. 3, the average body mass index (BMI) of patients at the baseline, first, second, and third month was 29.06 ± 0.69, 28.62 ± 0.68, 28.36 ± 0.68, and 27.98 ± 0.67, respectively, and the difference in BMI between patient visits was statistically significant (*P* < 0.001).

**Fig. 4:**
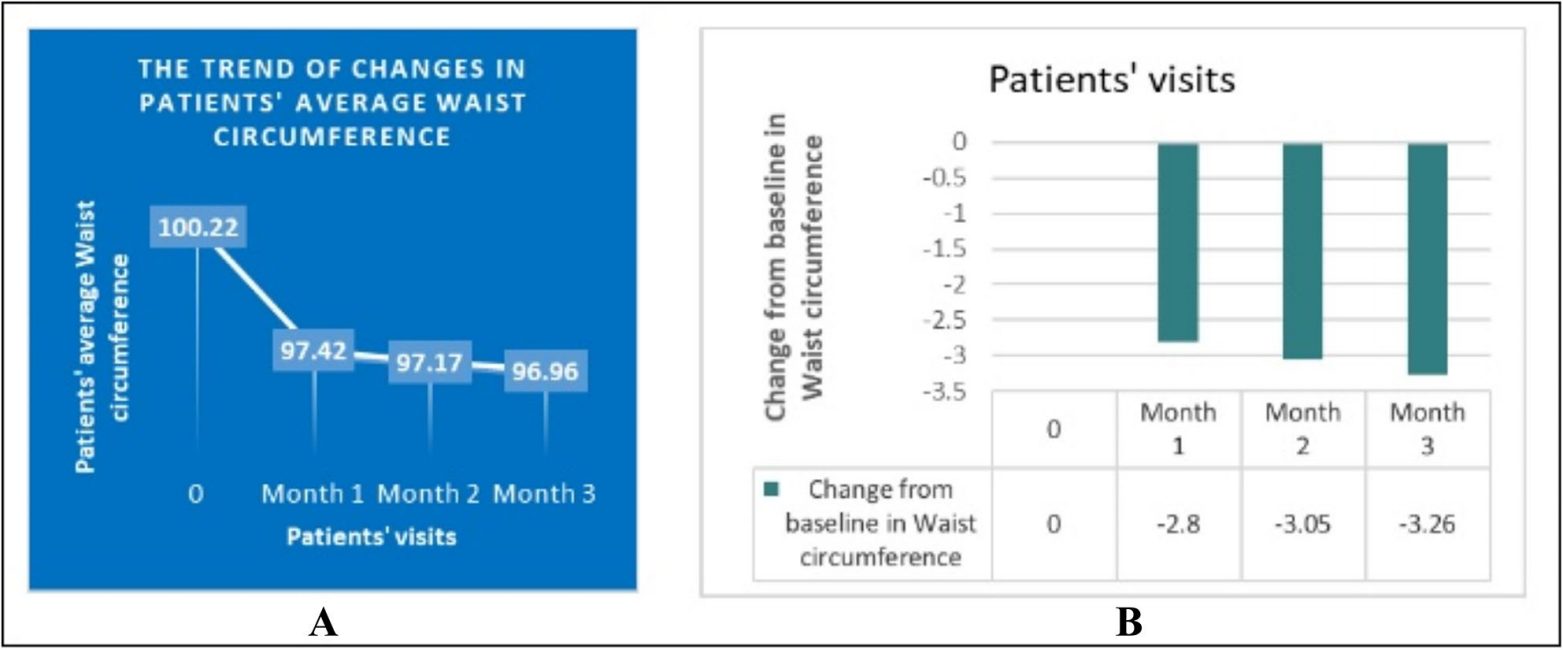
(**A**) The trend of changes in patients’ average Waist circumference. (**B**) Change from baseline in Waist circumference. Difference between baseline and first month =-2.806 cm (*P* value < 0.001). Difference between first and second month =-0.250 cm (*P* value = 0.655). Difference between second and third month =-0.208 cm (*P * value = 0.655).

According to Fig. 4, the average waist circumference of patients at the baseline, first, second, and third month was 100.22 ± 1.44, 97.41 ± 1.42, 97.16 ± 1.54, and 96.95 ± 1.42, respectively, and the trend of changes in waist circumference was also statistically significant, reaching a plateau after one month (*P* < 0.001).

To investigate the impact of weight change on other variables, Generalized Estimating Equations (GEE) were employed, revealing no significant correlation between weight variations and other laboratory-based data.

## Discussion

This semi-experimental clinical trial investigates the effects of 10 milligrams of Empagliflozin once daily as a first-line therapy in patients with prediabetes or diabetes with HbA1c levels up to 1% above the diagnostic threshold over 12 weeks. In this study, empagliflozin led to reductions in weight, body mass index (BMI), waist circumference, Fasting plasma glucose (FPG), and HbA1c levels. However, it did not significantly affect blood pressure. The weight loss was greater in men than women, but no significant differences were observed in the BMI or waist circumference changes between the two groups. The weight reduction was greater in patients with a glomerular filtration rate (GFR) above 90 compared to those with a GFR below 90. However, the two groups found no significant differences in the BMI or waist circumference changes. Furthermore, there were no significant differences in weight, BMI, or waist circumference changes among the different BMI groups. The trend of weight and BMI changes during the three-month treatment period with empagliflozin showed a significant and decreasing pattern, which did not reach a plateau after three months. A similar significant trend was observed in waist circumference, reaching a plateau after one month.

In the current study, the average weight of the patients decreased by 2.96 kg (3.8%) and the body mass index (BMI) fell by 1.10 Kg/m² (3.72%). The findings of this study are consistent with earlier research showing that empagliflozin promotes weight loss, although the amount of weight reduction differs between studies. Many studies indicate that using empagliflozin results in a mild weight reduction (less than 3.2%)^8–11^, and achieving greater weight loss necessitates long-term administration^12–15^. In contrast, some studies have shown more pronounced weight loss over shorter periods^16,17^, aligning with the findings of our research. For example, In the study by Mazhar Hussain et al. in Pakistan, patients experienced a significant weight reduction of -2.9 ± 6.4 kg after 12 weeks of treatment^18^.

In animal studies, this discrepancy is also observed. For example, in Bernhard Radlinger’s study in 2022, empagliflozin was found to protect mice from weight gain due to a high-calorie diet^19^ and in B Gaborit’s study on mice, a significant weight reduction was observed in response to empagliflozin intake at week 4^20^. However, Jolanda Sabatino et al. did not observe significant differences in weight after six weeks of empagliflozin treatment in mice^21^.

Empagliflozin promotes weight loss through various mechanisms. In the initial weeks, empagliflozin may lead to weight reduction by decreasing intravascular and extravascular fluid volumes. Empagliflozin promotes calorie excretion through glucosuria in the long term. This process leads to a reduction in body adipose tissue. Additionally, the weight loss observed following the use of empagliflozin in individuals with impaired kidney function indicates that other pathways for weight reduction exist; the mechanisms of these pathways are still unknown. In contrast, the weight loss following the use of empagliflozin is less than what would be expected due to glucosuria. Therefore, compensatory mechanisms, such as increased appetite and food intake, likely impede additional weight loss^22,23^. These findings suggest that genetic factors, dietary habits, and lifestyle may play a role in the observed variation in weight loss response to empagliflozin.

This difference was also reflected in the reduction of waist circumference. The reduction in waist circumference in our study was greater compared to previous studies^9,10^ and closely resembled the findings of another study conducted in Iran by Hooshmand Gharabagh et al.^16^.

In the current study, both weight and BMI exhibited a significant downward trend that did not plateau by the end of the study. Similarly, a notable reduction was observed in waist circumference, which stabilized after the first month. This finding is consistent with previous studies, which have demonstrated that weight loss continues until the sixth month and subsequently reaches a plateau^15,17^. The absence of weight stabilization in our study is likely due to the short follow-up period.

In our study, consistent with prior research^26,27^, greater weight reduction was observed in individuals with higher BMI. However, this difference did not reach statistical significance, likely due to the limited sample size.

Additionally, the weight reduction was greater in patients with a GFR above 90 compared to those with a GFR below 90, although no significant differences were found in the changes in BMI or waist circumference between the two groups. Similarly, David Z.I. Cherney et al. found that weight reductions with empagliflozin tended to decrease with decreasing baseline GFR^28^. These findings align with the mechanism of action of Empagliflozin. Empagliflozin works by inhibiting sodium-glucose co-transporter 2 (SGLT2) in the kidneys, promoting glucose excretion through urine (glucosuria). Patients with higher GFR experience more effective glucose filtration and excretion^29^, leading to greater caloric loss and, consequently, more pronounced weight reduction.

Limitations of our study include.

- Small sample size.
- Absence of a control group.
- Relatively short duration of patient follow-up.

Future studies should consider including control groups and conducting longer-term investigations. Given the influence of GFR on weight, patients with different GFR levels should also be investigated. Furthermore, it is recommended that studies on weight and weight-related indices be conducted in other countries with varying dietary habits.

## Conclusions

The results of this study indicated that the weight, body mass index (BMI), waist circumference, FPG, and HbA1c of patients decreased following the consumption of empagliflozin. The weight reduction was more pronounced in men than women and in individuals with a glomerular filtration rate (GFR) above 90 compared to those with a GFR below 90. The trend of changes in weight and BMI during the three-month treatment with empagliflozin was statistically significant and did not reach a plateau after three months. The trend of changes in waist circumference was also statistically significant and reached a plateau after one month.

## Data Availability

The datasets analyzed during the current study are available in the Harvard Dataverse repository, https://dataverse.harvard.edu/file.xhtml? fileId=10135470&version=1.0.

## Abbreviations

ANOVA: Analysis of Variance
BMI: Body Mass Index
cm: centimeter
DM: Diabetes Mellitus
FPG: Fasting plasma glucose
GFR: Glomerular Filtration Rate
HbA1c: Hemoglobin A1C
IRCT: Iranian Registry of Clinical Trials
Kg: kilogram
Kg/m²: kilograms per square meter
mmHg: millimeters of mercury
SGLT2 inhibitors: Sodium-Glucose Cotransporter-2 inhibitors
SPSS: Statistical Package for the Social Sciences
WHO: World Health Organization

## References

[1] Loscalzo, J. et al. Harrison’s Principles of Internal Medicine, 21 Edition. *Harrison’s Principles of Internal Medicine, 21e* (2022).

[2] Diabetes *World Health Organization (WHO)* https://www.who.int/health-topics/diabetes#tab=tab_1

[3] Diabetes. *World Health Organization (WHO)* https://www.who.int/news-room/fact-sheets/detail/diabetes

[4] Diabetes Iran (Islamic Republic of. ) 2016 country profile. https://www.who.int/publications/m/item/diabetes-irn-country-profile-iran-(islamic-republic-of)-2016

[5] Najafipour, H. et al. Prevalence and incidence rate of diabetes, pre-diabetes, uncontrolled diabetes, and their predictors in the adult population in Southeastern Iran: findings from KERCADR Study. *Front. Public. Health* **9**, (2021). 10.3389/fpubh.2021.611652 PMC8591105 34790639

[6] Najafipour, H. et al. Epidemiology of diabetes mellitus, pre-diabetes, undiagnosed and uncontrolled diabetes and its predictors in general population aged 15 to 75 years: A community-based study (KERCADRS) in southeastern Iran. *J. Diabetes*. **7**, 613–621 (2015). 25042896 10.1111/1753-0407.12195

[7] B Ortega, F., J Lavie, C. & N Blair, S. Obesity and cardiovascular disease. *Circul. Res.* **118**, Preprintathttpsdoiorg101161CIRCRESAHA115306883 (2016). 10.1161/CIRCRESAHA.115.306883 27230640

[8] Lazzaroni, E. et al. Anti-diabetic drugs and weight loss in patients with type 2 diabetes. *Pharmacological Research* vol. 171 Preprint at (2021). 10.1016/j.phrs.2021.105782 10.1016/j.phrs.2021.105782 34302978

[9] Neeland, I. J. et al. Empagliflozin reduces body weight and indices of adipose distribution in patients with type 2 diabetes mellitus. *Diab Vasc Dis. Res.* **13**, (2016). 10.1177/1479164115616901 PMC4768401 26873905

[10] Häring, H. U. et al. Empagliflozin as add-on to metformin plus sulfonylurea in patients with type 2 diabetes: A 24-week, randomized, double-blind, placebo-controlled trial. *Diabetes Care* **36**, (2013). 10.2337/dc12-2673 PMC3816918 23963895

[11] Kovacs, C. S. et al. Empagliflozin improves glycaemic and weight control as add-on therapy to pioglitazone or pioglitazone plus metformin in patients with type 2 diabetes: a 24-week, randomized, placebo-controlled trial. *Diabetes Obes. Metab.* **16**, (2014). 10.1111/dom.12188 23906415

[12] Ku, E. J., Lee, D. H., Jeon, H. J. & Oh, T. K. Empagliflozin versus dapagliflozin in patients with type 2 diabetes inadequately controlled with metformin, glimepiride and dipeptidyl peptide 4 inhibitors: A 52-week prospective observational study. *Diabetes Res. Clin. Pract.* **151**, (2019). 10.1016/j.diabres.2019.04.008 30954510

[13] Yabe, D. et al. Efficacy and safety of the sodium-glucose co-transporter-2 inhibitor empagliflozin in elderly Japanese adults (≥ 65 years) with type 2 diabetes: A randomized, double-blind, placebo-controlled, 52-week clinical trial (EMPA-ELDERLY). *Diabetes Obes. Metab.* **25**, (2023). 10.1111/dom.15249 37622398

[14] Rosenstock, J. et al. Improved glucose control with weight loss, lower insulin doses, and no increased hypoglycemia with empagliflozin added to titrated multiple daily injections of insulin in obese inadequately controlled type 2 diabetes. in *Diabetes Care* vol. 37 (2014). 10.2337/dc13-3055 24929430

[15] Tentolouris, A., Vlachakis, P., Tzeravini, E., Eleftheriadou, I. & Tentolouris, N. SGLT2 inhibitors: A review of their antidiabetic and cardioprotective effects. *International Journal of Environmental Research and Public Health* vol. 16 Preprint at (2019). 10.3390/ijerph16162965 10.3390/ijerph16162965 PMC6720282 31426529

[16] Hooshmand Gharabagh, L., Shargh, A., Mohammad Hosseini Azar, M. R. & Esmaeili, A. Comparison between the effect of empagliflozin and pioglitazone added to metformin in patients with type 2 diabetes and nonalcoholic fatty liver disease. *Clin. Res. Hepatol. Gastroenterol.* **48**, (2024). 10.1016/j.clinre.2023.102279 38159676

[17] Rodbard, H. W. et al. Oral semaglutide versus empagliflozin in patients with type 2 diabetes uncontrolled on metformin: The PIONEER 2 trial. *Diabetes Care* **42**, (2019). 10.2337/dc19-0883 31530666

[18] Hussain, M. et al. 2 diabetic patients. *J. Ayub Med. Coll.* **33**, (2021). 34137526

[19] Radlinger, B. et al. Empagliflozin protects mice against diet-induced obesity, insulin resistance and hepatic steatosis. *Diabetologia* **66**, (2023). 10.1007/s00125-022-05851-x PMC9947060 36525084

[20] Gaborit, B. et al. Effect of empagliflozin on ectopic fat stores and myocardial energetics in type 2 diabetes: The EMPACEF study. *Cardiovasc. Diabetol.* **20**, (2021). 10.1186/s12933-021-01237-2 PMC7919089 33648515

[21] Sabatino, J. et al. Empagliflozin prevents doxorubicin-induced myocardial dysfunction. *Cardiovasc. Diabetol.* **19**, (2020). 10.1186/s12933-020-01040-5 PMC7229599 32414364

[22] Johnston, R. et al. Canagliflozin, dapagliflozin and empagliflozin monotherapy for treating type 2 diabetes: Systematic review and economic evaluation. *Health Technol. Assess. (Rockv)* **21**, (2017). 10.3310/hta21020 PMC5292646 28105986

[23] Herrington, W. G. et al. Cardiac, renal, and metabolic effects of sodium–glucose co-transporter 2 inhibitors: A position paper from the European Society of Cardiology ad-hoc task force on sodium–glucose co-transporter 2 inhibitors. *Eur. J. Heart Fail.* **23**, (2021). 10.1002/ejhf.2286 34184823

[24] Tanaka, A. et al. Blood pressure reduction with empagliflozin in Japanese patients with type 2 diabetes and cardiovascular diseases: A post-hoc sub-analysis of the placebo-controlled randomized EMBLEM trial. *Hypertens. Res.* **47**, 2295–2302 (2024). 38789538 10.1038/s41440-024-01725-4

[25] Cheng, L. et al. Effect of SGLT-2 inhibitor, empagliflozin, on blood pressure reduction in Chinese elderly hypertension patients with type 2 diabetes and its possible mechanisms. *Sci. Rep.* **12**, (2022). 10.1038/s41598-022-07395-x PMC8894447 35241720

[26] Anker, S. D. et al. Weight change and clinical outcomes in heart failure with reduced ejection fraction: Insights from EMPEROR-Reduced. *Eur. J. Heart Fail.* **25**, (2023). 10.1002/ejhf.2728 PMC10098519 36325584

[27] Inzucchi, S. E. et al. Empagliflozin treatment effects across categories of baseline HbA1c, body weight and blood pressure as an add-on to metformin in patients with type 2 diabetes. *Diabetes Obes. Metab.* **23**, (2021). 10.1111/dom.14234 PMC7839733 33084149

[28] Cherney, D. Z. I. et al. Pooled analysis of Phase III trials indicate contrasting influences of renal function on blood pressure, body weight, and HbA1c reductions with empagliflozin. *Kidney Int.* **93**, 231–244 (2018). 28860019 10.1016/j.kint.2017.06.017

[29] Zanoli, L. et al. Sodium-glucose linked transporter-2 inhibitors in chronic kidney disease. *Scientific World Journal* vol. 2015 Preprint at (2015). 10.1155/2015/317507 10.1155/2015/317507 PMC4345065 25785281

